# Prevalence and Association of Liver Steatosis and Non-Alcoholic Pancreatic Steatosis in Very High Cardiovascular Risk

**DOI:** 10.3390/diseases14050154

**Published:** 2026-04-28

**Authors:** Raúl Gómez-Mendoza, Eva Juárez-Hernández, Vicente Toledo-Coronado, César A. Tenorio-Aparicio, Javier Sánchez-Zavala, Misael Uribe, Graciela Castro-Narro, Iván López-Méndez

**Affiliations:** 1 Gastroenterology and Obesity Unit, Medica Sur, Mexico City 14050, Mexico; raul.93gome@gmail.com (R.G.-M.); muribe@medicasur.org.mx (M.U.); 2 Translational Research Unit, Medica Sur, Mexico City 14050, Mexico; evajuarezh@hotmail.com; 3 Imaging Unit, Medica Sur, Mexico City 14050, Mexico; drvicentetoledo@gmail.com (V.T.-C.); c.tenorio124@gmail.com (C.A.T.-A.); 4 Coronary Care Unit, Medica Sur, Mexico City 14050, Mexico; jsanchezz@medicasur.org.mx; 5 Transplants and Hepatology Unit, Medica Sur, Mexico City 14050, Mexico; gracastron@hotmail.com

**Keywords:** steatosis, liver, pancreas, cardiovascular risk

## Abstract

Background/Objectives: In the last decade, the prevalence of metabolic-associated fatty liver disease (17–46%) and non-alcoholic fatty pancreas disease (NAFPD) (16–33%) has increased due to their association with obesity, both predictors of early atherosclerosis and metabolic risk. Computed tomography (CT) has been proposed as a diagnostic method. Currently, the factors associated with NAFPD have not been fully described. The aim of this study is to describe the prevalence and association of NAFPD and liver steatosis in patients with very high cardiovascular risk. Methods: A retrospective evaluation was conducted on the medical records of patients classified as very high cardiovascular risk who had undergone a CT scan. NAFPD was determined by the difference in pancreatic and splenic attenuation (−1.9), while liver steatosis was identified by hepatic attenuation <40. Bivariate and multivariate analyses were performed to determine the independent factors associated with NAFPD. Results: 169 medical records were collected; 68.6% (*n* = 116) were men, with a median age of 70 [IQR 61–78] years and 25.8 [IQR 23.7–28.7] kg/m^2^ of body mass index. According to the CT scans, 80.5% (*n* = 136) presented NAFPD, 24.3% (*n* = 41) had liver steatosis, and 21.3% (*n* = 36) had both. In the multivariate analysis, liver steatosis, abnormal levels of aspartate aminotransferase, and being overweight were independent factors associated with NAFPD. Conclusions: In a very high cardiovascular-risk population, the prevalence of NAFPD is high, and it is independently associated with the presence of liver steatosis.

## 1. Introduction

Pancreatic steatosis was described for the first time in 1926 [1]. Since then, different terms have been suggested to describe the accumulation of fat in the pancreas, or pancreatic steatosis. One of them is Non-alcoholic Fatty Pancreas Disease (NAFPD), which is used to describe a subtype of pancreatic steatosis associated with obesity and metabolic syndrome (MetS), in the absence of excessive alcohol consumption [2].

The pathophysiology of NAFPD is associated with a positive energy balance, resulting in the accumulation of free fatty acids in various organs, including the liver, heart, and vascular endothelium. This excess fat triggers the release of proinflammatory cytokines, promoting systemic inflammation and insulin resistance (IR) [3]. The prevalence of NAFPD in the adult population has been estimated between 16% and 33% [4]. The primary factors associated with the accumulation of pancreatic fat include MetS, age, and the presence of Metabolic Dysfunction-Associated Steatotic Liver Disease (MASLD) [5]. The latter is considered part of the MetS spectrum [6,7] and has gained significant importance due to its risk of progressing to hepatic fibrosis and its association with extrahepatic diseases such as type 2 diabetes mellitus (DM) and cardiovascular diseases [5,8,9]. Currently, it is recognized as the foremost cause of chronic liver disease worldwide. [8,10]. NAFPD has been linked to an increased risk of developing arterial hypertension (OR 1.67; 95% CI, 1.32–2.10; *p* < 0.0001), DM (OR 2.08; 95% CI, 1.44–3.00; *p* = 0.0001), and MetS (OR 2.37; 95% CI, 2.07–2.71; *p* < 0.0001) [11,12].

The reference standard for the diagnosis of pancreatic steatosis is detecting pancreatic fat ≥6.2% through histology [13]; likewise, the diagnosis of liver steatosis is confirmed with >5% of fat in a biopsy of hepatic tissue [10]. However, the invasive nature of biopsy limits its use in clinical practice; therefore, non-invasive imaging techniques, such as computed tomography (CT), have become the preferred alternative and have been shown to correlate well with histopathology in both scenarios. For pancreatic steatosis detection by CT, a cutoff value of −1.9 of the attenuation-based index, which estimates the difference between pancreatic and splenic attenuation (P-S), has a sensitivity of 80% and a specificity of 42%; on the other hand, the pancreas-spleen attenuation ratio (P/S), with a cutoff value of 0.9, shows a sensitivity of 70% and specificity of 58% [13,14].

Regarding the determination of liver steatosis, CT has demonstrated high performance in detecting liver steatosis compared to biopsy, with a sensitivity of 72% and a specificity of 88% [15].

The clinical significance of diagnosing NAFPD lies in its association with other diseases, such as chronic pancreatitis (OR 3.96; 95% CI, 2.04–7.66) [16], pancreatic cancer (OR 18.027; 95% CI, 7.28–44.58) [17], alterations in insulin secretion, and DM [12], as well as MASLD [18]. Furthermore, its association with MetS and an increased cardiovascular risk (CVR) has been reported [12,19]. A recent meta-analysis that included 49,329 patients found that NAFPD was significantly associated with an increased risk of MetS (RR = 2.25; 95% CI, 2.00–2.53; *p* < 0.0001), central obesity (RR = 1.91; 95% CI, 1.67–2.19; *p* < 0.0001) and MASLD (RR = 2.49; 95% CI, 2.06–3.02; *p* < 0.0001) [20]. Regarding CVR, the leading cause of mortality in patients with MetS and MASLD, a study by Kul et al. [21] found that epicardial adipose tissue, a marker of ectopic fat, and aortic intima-media thickness, an indirect indicator of subclinical atherosclerosis, were significantly higher in subjects with NAFPD.

Since 2018, the European Society of Cardiology, the European Atherosclerosis Association and the American Heart Association have proposed the “very high cardiovascular risk” definition, which includes patients with a history of one major atherosclerotic cardiovascular disease: recent acute coronary syndrome, a history of myocardial infarction, a history of ischemic stroke, or symptomatic peripheral arterial disease in addition to at least one high-risk condition such as age > 65 years, familial hypercholesterolemia, a history of prior artery bypass surgery, DM, hypertension, chronic kidney disease, current smoking, persistently elevated low-density lipoprotein (despite treatment with statin and ezetimibe), and a history of congestive heart failure [22,23]. These patients are characterized by a persistent inflammatory status, which, in combination with metabolic abnormalities, may lead to an increased risk of liver and pancreatic steatosis [24,25].

Despite the increasing evidence suggesting that NAFPD may negatively impact metabolism, predisposing individuals to MetS, MASLD, and the development of subclinical atherosclerosis [26]. The role of pancreatic steatosis remains an ongoing debate since there is evidence that suggests no relationship between pancreatic-liver steatosis and CVR. Specific risk factors for pancreatic steatosis have not been fully described; therefore, evaluating populations with very high CVR, such as patients in coronary care units, could provide evidence on the prevalence of this entity and its relationship with CVR, as well as comparing it with liver steatosis prevalence. This study aims to describe the prevalence and association of NAFPD and liver steatosis in patients with very high CVR.

## 2. Materials and Methods

### 2.1. Study Population

**Figure 1:**
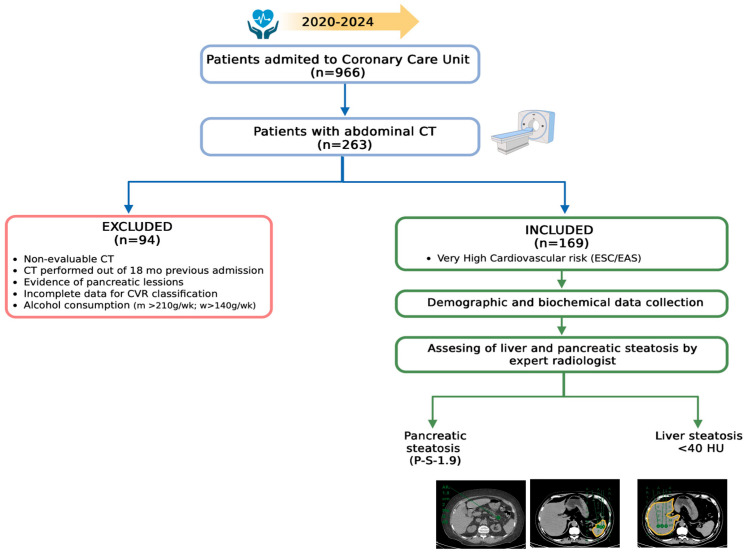
Diagram of selection criteria. CT Computed Tomography; CVR Cardiovascular Risk; m men; w woman; ESC European Society of Cardiology; EAS European Atherosclerosis Society; P-S Difference between pancreatic and splenic attenuation; HU Hounsfield units. Created in BioRender. Juarez-Hernández, E. (2025). https://BioRender.com/li8d2vn.

This is a retrospective medical record revision of adult patients (>18 years) admitted to the Coronary Care Unit of a tertiary care private hospital in Mexico City from 2020 to 2024 that met criteria for group 1 of very high CVR: documented clinical atherosclerotic cardiovascular disease, according to the European Society of Cardiology and the European Atherosclerosis Society [23], and had a non-contrast abdominal CT scan performed during their admission or within 18 months prior to the cardiovascular event for any reason (Figure 1).

Biochemical data of blood glucose, lipids, and liver function tests were recorded and dichotomized according to abnormal values as follows: blood glucose > 100 mg/dL; triglycerides > 150 mg/dL; high-density lipoprotein (HDL) < 40 mg/dL for men and < 60 mg/dL for women; cholesterol > 200 mg/dL, aspartate aminotransferase (AST) > 47 U/L; alanine aminotransferase (ALT) > 40 U/L; and gamma-glutamyl transpeptidase (GGT) > 50 U/L. The exclusion criteria were non-evaluable CT images, evidence of pancreatic lesions, and incomplete data for CVR assessment; also, records of patients with excessive alcohol consumption (>210 g/week for men and >140 g/week for women), as referred to in the medical records, were excluded (Figure 1).

### 2.2. MASLD and NAFPD Assessment

Radiologists from the diagnostic imaging service reviewed the CT images to exclude pancreatic lesions, ensuring the integrity of the pancreatic parenchyma and vascular structures. A single expert radiologist assessed the presence of pancreatic steatosis using the attenuation index, based on both contrast-enhanced and non-contrast CT images. Three regions of interest (ROIs) with areas of 1.0 cm^2^ in different pancreatic sections were selected using a dedicated 3D workstation. The mean of pancreatic attenuation from the three ROI measurements was considered representative for each subject. We used the average of three cm^2^ ROIs from different spleen areas on non-enhanced CT images for the splenic attenuation. A cutoff value of −1.9 for P-S and a cutoff value of 0.9 for P/S were used to detect pancreatic steatosis. Meanwhile, liver steatosis was assessed using non-contrast abdominal tomography, where liver steatosis was defined as a hepatic attenuation of <40 Hounsfield units.

### 2.3. Statistical Analysis

The frequency and percentage of categorical variables, as well as the median and interquartile ranges (IQR) of continuous data, were estimated for descriptive analysis.

Given the high prevalence of NAFPD (80.5%), Prevalence Ratios (PR) and 95% confidence intervals were estimated using Poisson regression with robust (sandwich) variance. The variables analyzed included gender; overweight (Body Mass Index (BMI) 25–29.9 kg/m^2^) and obesity (BMI ≥ 30 kg/m^2^); presence of DM; high blood pressure; dyslipidemia, as documented in medical records of diagnosis or treatment; history of smoking; and dichotomized biochemical values. (glucose, triglycerides, HDL, LDL, total cholesterol, AST, ALT, and GGT) Variables with *p* < 0.10 in the univariate analysis were included in the multivariate model; *p* < 0.05 was considered statistically significant. Model calibration was evaluated using the Hosmer–Lemeshow test, and discrimination was assessed using the area under the ROC curve (AUC). Collinearity was assessed using the Variance Inflation Factor. Statistical analyses were performed using IBM SPSS Statistics, version 21.0. 

## 3. Results

A total of 169 medical records met the inclusion criteria and were included in the analysis. 68.6% (*n* = 116) were male, with a median age of 70 [IQR 61–78] years and 25.8 [IQR 23.7–28.7] kg/m^2^ of BMI. DM was present in 32% (*n* = 54) of the patients; 60.9% (*n* = 103) had hypertension, 26.6% (*n* = 45) had dyslipidemia, and 26.6% (*n* = 45) had a history of smoking. According to BMI, 39.6% (*n* = 67) of patients were overweight, and obesity was present in 19.5% (*n* = 33). Regarding biochemical variables, the medians of glucose and triglycerides were 109 [IQR 96–134] mg/dL and 112 [IQR 84–144] mg/dL, respectively. The general characteristics of the patients are presented in Table 1.

Regarding previous history of cardiovascular events, 30.8% (*n* = 52) had a history of acute myocardial infarction, 3.6% (*n* = 6) had experienced a cerebrovascular event, 39.6% (*n* = 67) had acute coronary syndrome, and 45.6% (*n* = 77) had ischemic cardiopathy.

**Figure 2:**
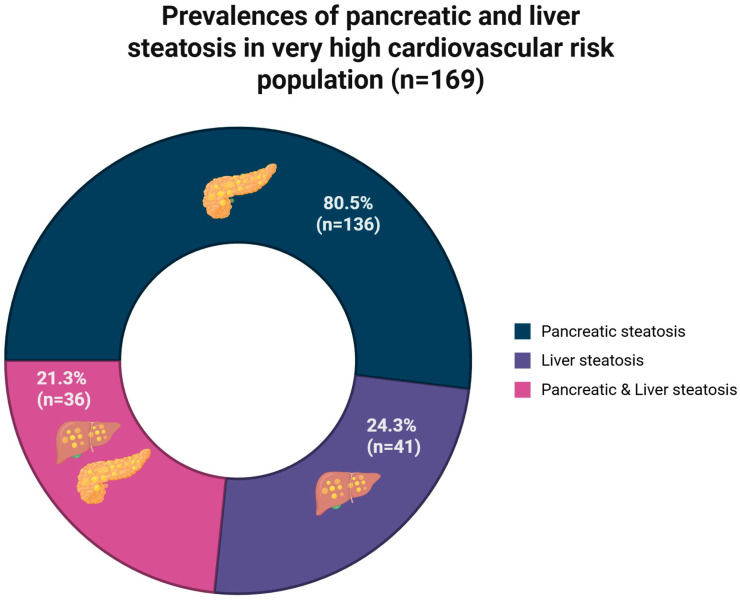
Prevalences of pancreatic steatosis, liver steatosis, and both. Created in BioRender. Juárez-Hernández, E. (2025). https://BioRender.com/k95v303.

According to CT, 80.5% (*n* = 136) had NAFPD (P-S as the diagnostic reference), 24.3% (*n* = 41) had liver steatosis, and 21.3% (*n* = 36) had both (Figure 2). Meanwhile, the prevalence of NAFPD was 61.9% under the P/S criteria.

In the univariate analysis, liver steatosis (OR 6.23, 95% CI 1.42–27.31; *p* = 0.006), overweight (OR 0.40, 95% CI 0.19–0.87; *p* = 0.028), and elevated AST > 47 U/L (OR 0.44, 95% CI 0.19–0.98; *p* = 0.047) showed significant associations with NAFPD and were included in the multivariate model (Table 2).

In the multivariate Poisson regression model, liver steatosis was independently associated with NAFPD (PR 1.21, 95% CI 1.07–1.36; *p* = 0.002), as was overweight (PR 0.83, 95% CI 0.71–0.98; *p* = 0.029). Elevated AST did not retain independent significance (PR 0.84, 95% CI 0.68–1.04; *p* = 0.114). The model showed adequate calibration (Hosmer–Lemeshow χ^2^(8) = 3.89, *p* = 0.867) and acceptable discrimination (AUC = 0.715).

The model was adjusted by BMI ≥ 25 kg/m^2^; in the Poisson regression model, only liver steatosis showed a significant independent association (PR 1.22 (95% CI 1.08–1.37; *p* = 0.001)); overweight and AST > 47 did not retain a significant association.

When univariate and multivariate analyses were performed using P/S as NAFPD diagnostic criteria (prevalence 61.9% (*n* = 104)); only overweight showed a significant association (OR 0.46, 95% CI 0.24–0.86; *p* = 0.08); however, the presence of DM (OR 1.88, 95% CI 0.93–3.80; *p* = 0.08) and smoking (OR 1.97, 95% CI 0.96–4.04; *p* = 0.08) were included in the multivariate analysis. Finally, DM (OR 1.30, 95% CI 1.04–1.62; *p* = 0.023) and overweight (OR 0.73, 95% CI 0.56–0.02; *p* = 0.020) were independently associated with NAFPD; however, discrimination of the model was moderate (AUC 0.627).

## 4. Discussion

In our study, we analyzed 169 patients admitted to the Coronary Care Unit, most of whom were men of advanced age. We found a high prevalence of pancreatic steatosis, and 21.3% of these cases coexist with liver steatosis.

The epidemiology of NAFPD has not been fully established due to the lack of specifically defined diagnostic criteria and methods; however, the prevalence of NAFPD in the USA has been estimated to be between 16% and 35% [4]. In a multicentric study, Sezgin et al. found a prevalence of 68.9% (*n* = 1700) in an outpatient reference clinic [27]. In our population, the prevalence of NAFPD was higher than previously reported (80.5%); however, it is essential to note that our study was conducted in a population with higher metabolic risk and does not consist of an obese population.

Nowadays, the metabolic implications of pancreatic steatosis are not fully understood; evidence shows an interaction between pancreatic steatosis and other organs, with effects on inflammatory signaling pathways. The recent study conducted by Antony et al. [28] included 37 patients who underwent a total or distal pancreatectomy due to evidence of pancreatic neoplasms. After twelve months of follow-up, it was observed that there was a significant increase in the incidence and severity of liver steatosis in patients who underwent a total pancreatectomy (*p* = 0.002). The authors conclude that the development of liver steatosis after pancreatic resections results from various factors, including pancreatic exocrine insufficiency, loss of duodenal hormones, portal endotoxemia, micronutrient deficiencies, and post-pancreatectomy diarrhea.

On the other hand, confirming the interaction between the pancreas and different organs and pathways, it is well known that patients with DM and obesity have impairments in β-pancreatic cell function, which is associated with IR. Oe et al. [29] conducted a study on patients with DM and obesity who underwent sleeve gastrectomy to determine the impact of bariatric surgery on β-pancreatic cell function. After one year of follow-up, a significant improvement was observed in the insulin secretion-sensitivity index-2 (0.91 ± 0.39 to 1.4 ± 0.7, *p* < 0.01). This finding highlights the interaction of the pancreas with multiple metabolic pathways.

In patients with very high CVR, the high prevalence of liver steatosis and NAFPD does not seem coincidental. Given the multifactorial etiology, insulin resistance, which drives excessive lipolysis in visceral adipose tissue, appears to be a key factor in the development of steatosis in both organs, with a continuous flow of free fatty acids to the liver and pancreas that simultaneously activates hepatic de novo lipogenesis and induces lipotoxic dysfunction of pancreatic β cells through the accumulation of diacylglycerols and ceramides [12,30].

The very high CVR in the presence of liver steatosis/NAFPD results from the synergistic convergence of these pathways—insulin resistance, lipotoxicity, hepatic atherogenic dyslipidemia, systemic inflammation, intestinal dysbiosis, and pancreatic dysfunction—which can lead to an exponential increase in CVR, highlighting the importance of both types of steatosis that can become potential therapeutic targets [31,32,33].

The results of these studies suggest that pancreatic steatosis is an underestimated entity that plays a role in regulating visceral fat accumulation. Probably, the presence of pancreatic steatosis is another factor involved in metabolic impairment, increasing liver steatosis, inflammation, and IR.

Regarding liver steatosis, in our study, the prevalence was also high; this elevated prevalence may be due to our sample comprising patients with very high CVR and a high prevalence of comorbidities, such as DM and hypertension. Additionally, 39.4% of the patients had a history of acute myocardial infarction, and 18.2% had heart failure conditions that contribute to a pro-inflammatory state and IR, which are associated with the development of NAFPD [4]. In a similar population, Yilmaz et al. [34] observed that liver steatosis, evaluated by ultrasound, could predict the presence and severity of coronary arterial diseases according to the Gensini score, showing significantly higher values between liver steatosis grades 0 and 2 and 3 (9.8 ± 11.9 vs. 56.7 ± 26.4, *p* ≤ 0.0001). These patients also exhibited significantly elevated levels of AST (27.0 ± 15.9 vs. 28.3 ± 12.3, *p* = 0.02). Despite these important results, liver steatosis was evaluated using ultrasound, a method that is operator-dependent and has lower diagnostic accuracy compared to CT [35].

The presence and progression of visceral fat accumulation, including hepatic and pancreatic steatosis, are attributed to a state of chronic low-grade inflammation that involves the production of proinflammatory cytokines in adipose tissue. In this process, it seems that splenic volume plays a significant role. The spleen has been associated with inflammatory diseases, but specifically in metabolic-inflammatory diseases such as obesity and liver steatosis, it seems that the spleen’s immune system has pathogenic connections between low-grade chronic inflammation, IR, and the development of liver steatosis due to shared metabolic activities of the liver-spleen axis; therefore, ultrasonography measurement of spleen volume has been proposed to improve liver steatosis diagnosis [36].

This study achieved NAFPD diagnosis through CT, using the P-S, which has a higher sensitivity than the P/S. Despite this, according to the P/S, the prevalence of NAFPD was lower (61.9%, *n* = 104). Although CT is not the standard diagnostic reference [37], it has been described that this technique correlates significantly with histological findings (r = −0.622, *p* < 0.01 for P-S) and also has been shown to have a strong inverse correlation with other image diagnostic methods such as magnetic resonance (Spearman coefficient −0.755) [38,39] regardless of patient factors, with the ability to evaluate the entire pancreas [40]. Therefore, it could be used to assess pancreatic fat in large population studies [14].

Cardiovascular disease is the leading cause of mortality in patients with MASLD; the underlying mechanism may involve metabolic dysfunction and an increased pancreatic fatty acid load in individuals with fatty liver, predisposing them to pancreatic fat accumulation, in a bidirectional way [3]; given this relationship, it is not unusual to assume that patients with NAPFD have an increased risk of cardiovascular alterations. In a study that included one hundred patients with MASLD, confirmed through liver biopsy, and thirty-four controls, Ozturk et al. observed significant correlations between the presence of fatty pancreas (evaluated by ultrasound) and higher carotid intima-media thickness and carotid-femoral pulse wave velocity; these correlations were maintained even after confounder adjustment [41]. In 324 liver steatosis patients, Marti-Aguado et al. [31] observed that pancreatic steatosis (evaluated by magnetic resonance imaging) was independently associated with a high risk of cardiovascular disease (OR: 3.15, 95% CI 1.63–6.09; *p* = 0.001). Regarding the very high CVR population, which includes DM patients, pancreatic fat has been proposed as an endocrine modulator of IR [42]; therefore, the presence of pancreatic steatosis has been related to an incidence of DM (adjusted OR per 10 Hounsfield units lower attenuation: 1.32 [95% CI 1.06–1.63], *p*  =  0.012) [43]. In a prospective cohort study of 42,599 patients, Dong et al. [44] observed that pancreatic fat, as quantified by magnetic resonance imaging, independently increases the risk of DM by 33.7%; furthermore, pancreatic fat also increases the risk of acute and chronic pancreatitis (298.2% and 97.6%, respectively). In patients with very high CVR, the presence of NAPFD could be an additional risk for increased severity or progression of MetS, as well as pancreatic abnormalities.

Under conditions of fatty acid excess, when subcutaneous adipose tissue reaches its storage capacity, fatty acids are deposited in ectopic sites, such as the pancreas, liver, and other organs. This ectopic fat triggers systemic inflammation and IR and correlates significantly, according to sex, with different biomarkers of MetS, such as triglycerides (r = 0.46 w r = 0.37 m), HDL (r = 0.35 w r = 0.33 m), and blood glucose (r = 0.34 w r = 0.19 m) [3,45]. Due to these correlations, NAFPD has been linked to MetS and obesity [2]. In our study, the prevalence of MetS in patients with NAFPD was 52.5% (*n* = 42/80); in addition to the relationship between ectopic fat accumulation and the development of metabolic diseases and their complications, the presence of pancreatic steatosis has been particularly associated with local complications such as chronic pancreatitis (OR 3.96 [95% CI 2.04, 7.66]) [16] and pancreatic cancer (OR 18.027 [95% CI 7.28–44.58]) [17].

All of the results provide evidence for the association between metabolic abnormalities and NAFPD. However, this association may deteriorate with the addition of CVR, a condition inherently linked to metabolic impairment. This situation is particularly pertinent in current epidemiological contexts characterized by the increasing prevalence of obesity. Overweight/obesity is linked to increased levels of inflammatory cytokines (interleukin-6 and C-reactive protein) and significantly reduced levels of adiponectin, which is associated with coronary artery calcification and stroke [46]. Inflammation represents a pivotal mechanism implicated in cardiovascular diseases and their associated comorbidities, including DM and metabolic syndrome MetS. Both obesity and cardiometabolic abnormalities appear to be significant determinants influencing the progression of pancreatic steatosis, thereby exacerbating pancreatic stiffness. Specifically, glucose impairments have been proposed as stronger predictors of elevated stiffness in patients with pancreatic fat infiltration [47].

One of the consistent findings of our study is the inverse association between overweight and NAFPD; this result could be associated with the average age of our patients (70 years), where sarcopenia and visceral redistribution of fat with age are prevalent [48]. BMI in the overweight range may represent a metabolically less adverse phenotype than normal weight or obesity, possibly due to better nutritional status and lower chronic systemic inflammation. However, BMI is a poor surrogate for visceral adiposity [49], as it is not a parameter that distinguishes between muscle mass and fat mass, so the measurement of ectopic pancreatic fat accumulation correlates more strongly with visceral adiposity than with BMI itself [50,51].

Our study has limitations, including its retrospective design and the methods used to detect steatosis. Notwithstanding, at this moment, there are no specific guidelines for the diagnosis of pancreatic steatosis, and the reference standard for diagnosis is a procedure that represents significant risks and complications for the patient (pancreatic biopsy). Although CT has shown good concordance with pancreatic and liver biopsy, other non-invasive methods have better performance, particularly magnetic resonance and transient elastography in liver steatosis [52], However, due to the retrospective nature of our study and the setting of the patients, the detection of pancreatic or liver steatosis was not an intentional focus in managing the clinical scenario. Despite the very high CVR nature of our population, the median values of blood lipids were within normal ranges. Even when the history of dyslipidemia showed a significant association with the presence of NAFPD in univariate analysis, this association was not maintained in the multivariate analysis. This could be explained by the fact that these patients could already be under pharmacological treatment, which is also a characteristic of patients classified as very high CVR.

Currently, the evidence regarding the association between pancreatic steatosis and CVR remains controversial. Consequently, this study aims to evaluate the factors associated with pancreatic steatosis in patients who have already been identified as having very high CVR, rather than investigating steatosis as a potential risk factor; our results show a significant association with liver steatosis in these patients. Future prospective studies are necessary to highlight the importance of pancreatic steatosis, not only as an additional CVR factor but also as a standalone disease that could be associated with pancreatic diseases, liver steatosis, and MetS.

It is imperative to acknowledge that the correlation between CVR and pancreatic steatosis remains controversial. Independent of the limitations, our results show a significant association between the presence of pancreatic and liver steatosis in patients with very high CVR, which supports the need for additional prospective studies to explain the role of NAFPD in both the general population and in patients with high metabolic risk more precisely.

Liver steatosis and CVR are significant targets for clinical trials; however, according to our results and the emerging evidence regarding NAFPD, this could be considered an additional pathology that deserves inclusion in trial designs. Although the evidence is limited, it appears to be the tip of the iceberg in describing a probable new metabolic pathway.

## 5. Conclusions

In a very high CVR population, NAFPD has a high prevalence, and it is independently associated with the presence of liver steatosis.

## Figures and Tables

**Table 1:** General characteristics of the population.

Characteristic	M [IQR]/% (*n*)
Male	68.6 (116)
Age (years)	70 (61–78)
BMI (kg/m^2^)	25.8 (23.7–28.7)
Overweight	39.6 (67)
Obesity	19.5 (33)
DM	32.3 (54)
HTN	61.7 (103)
Dyslipidemia	26.9 (45)
Smoking	31.7 (53)
Blood glucose (mg/dL)	109 (96–134)
Triglycerides (mg/dL)	112 (84–144)
HDL (mg/dL)	38 (33–45)
LDL (mg/dL)	78 (60–98.5)
Cholesterol (mg/dL)	140 (121.5–165.5)
AST (U/L)	26 (21–47)
ALT (U/L)	28 (20–42)
GGT (U/L)	31 (21–49)

BMI, Body Mass Index; DM, Diabetes Mellitus; HTN, Hypertension; HDL, High-Density Lipoprotein; LDL, Low-Density Lipoprotein; AST, Aspartate Aminotransferase; ALT, Alanine Aminotransferase; GGT, Gamma Glutamyl Transpeptidase.

**Table 2:** Univariate and multivariate analysis of factors associated with NAPFD.

Factor	Univariate		Multivariate Poisson Regression Model	
	OR (95% CI)	*p*	PR (95% CI)	*p*
Male	1.32 (0.6–2.9)	0.53		
DM	1.81 (0.73–4.71)	0.28		
HTN	1.2 (0.54–2.66)	0.68		
Dyslipidemia	2.17 (0.78–6.04)	0.17		
Smoking	1.75 (0.7–4.37)	0.28		
Overweight (BMI 25–29.9)	0.4 (0.19–0.87)	0.02	0.83 (0.71–0.98)	0.02
Obesity	1.11 (0.42–2.97)	1.00		
BMI ≥ 25	0.39 (0.17–0.93)	0.03		
TG > 150	1.17 (0.44–3.1)	1.00		
Low HDL *	0.73 (0.32–1.71)	0.53		
LDL > 100	0.75 (0.32–1.8)	0.49		
TC > 200	0.56 (0.2–1.57)	0.25		
AST > 47	0.44 (0.19–0.98)	0.04		
ALT > 40	0.8 (0.35–1.84)	0.66		
GGT > 50	1.38 (0.53–3.65)	0.64		
Liver steatosis	6.23 (1.42–27.3)	0.005	1.21 (1.07–1.36)	0.001

DM, Diabetes Mellitus; HTN, Hypertension; BMI, Body Mass Index; TG, Triglycerides; HDL, High-Density Lipoprotein; LDL, Low-Density Lipoprotein; TC, Total Cholesterol; AST, Aspartate Aminotransferase; ALT, Alanine Aminotransferase; GGT, Gamma-Glutamyl Transpeptidase; * according to gender.

## Data Availability

The data presented in this study are available on request from the corresponding authors. The data are not publicly available due to privacy and ethics.

## Abbreviations

The following abbreviations are used in this manuscript:

NAFPD	Nonalcoholic Fatty Pancreas Disease
MetS	Metabolic Syndrome
IR	Insulin Resistance
MASLD	Metabolic Dysfunction-Associated Steatotic Liver Disease
DM	Type 2 Diabetes Mellitus
CT	Computed Tomography
P-S	Pancreatic and Splenic attenuation
P/S	Pancreas/spleen attenuation ratio
CVR	Cardiovascular Risk
HDL	High Density Lipoprotein
AST	Aspartate Aminotransferase
ALT	Alanine Aminotransferase
GGT	Gamma-Glutamyl Transpeptidase
ROI	Regions of Interest
IQR	Interquartile Range
BMI	Body Mass Index
